# Early Deaths after Arterial Ischemic Stroke in Pediatric Patients: Incidence and Risk Factors

**DOI:** 10.3390/children8060471

**Published:** 2021-06-03

**Authors:** Ilona Kopyta, Agnieszka Cebula, Beata Sarecka-Hujar

**Affiliations:** 1 Department of Paediatric Neurology, Faculty of Medical Sciences in Katowice, Medical University of Silesia in Katowice, Medykow Str 16, 40-752 Katowice, Poland; ilonakopyta@autograf.pl (I.K.); cebula.ap@gmail.com (A.C.); 2 Department of Basic Biomedical Science, Faculty of Pharmaceutical Sciences in Sosnowiec, Medical University of Silesia in Katowice, Kasztanowa Str 3, 41-200 Sosnowiec, Poland

**Keywords:** arterial ischemic stroke, children, mortality, death, outcome

## Abstract

In developed countries, cerebrovascular diseases are among the 10 most common causes of death in both the pediatric and adult population. The prevalence of fatal outcomes following arterial ischemic stroke (AIS) in various groups of pediatric patients ranges from 1% to almost 32%. However, a constant improvement in stroke mortality among children has been observed. The extent of the decline differs among studies (from nearly tenfold to twofold decline), as it depends on the study population. While a portion of this variability might be explained by factors such as health care access, population age, diseases related to ethnicity, and different etiologies of stroke in studied populations, the understanding of such differences is still insufficient. Risk factors for death in the early stages of the disease are poorly understood and are usually based on the clinical presentations of relatively small groups of pediatric patients. Familiarity with these factors may be of significant importance for prognosis, but also for the early selection of patients requiring careful supervision. The present study aimed to analyze and discuss the current literature data on the incidence of early death and risk factors for early death in children suffering from stroke.

## 1. Introduction

Arterial ischemic stroke (AIS) has a multifactorial origin both in children and adults. In developed countries, cerebrovascular diseases are among the 10 most common causes of death [1].

The prevalence of AIS in children is reported as approximately three new cases per year per 100,000 children. In turn, AIS in adulthood affects about 15% of young patients [2]. The risk factors that predispose the pediatric population to AIS differ between children and neonates and from those observed in the adult population [3,4,5,6].

Long-term outcomes affect most children after stroke and require many years of specialized care and support for development [7]. The problem of death in children with stroke is poorly understood, especially the aspect of predisposing factors. However, constant improvement in stroke mortality among children has been observed. Among the reasons are a gradual decline of risk factors, advances in diagnostics, new treatment options, and progress made in intensive care. This tendency has been observed in the pediatric population since the 1950s, as described by Mallick et al. [8] in their study on stroke mortality in England and Wales in 1920–2000. Fullerton et al. [9] demonstrated a decline in stroke mortality for children from 0.1 to 0.08 per 100,000 person-years between 1979 and 1998. At the same time, data published by the Global and Regional Burden of Stroke 2013 Experts Group showed that even though there was a clear increase in the absolute number of pediatric stroke cases between 1990 and 2013, there was a two-fold decline in stroke-related mortality [10].

Since stroke is a medical emergency, all patients who experience AIS are at a very high risk of death both during the acute phase and during follow-up. Knowledge of risk factors for post-stroke death in the first days after the acute event is especially needed. These factors can be divided into those related to the AIS itself, i.e., malignant brain edema, herniation, massive hemorrhagic, transformation of ischemic focus, and size of ischemic focus, and those related to therapeutic procedures and comorbidities [11]. Such knowledge could influence the prevention and management of stroke, which in turn could improve the long-term survival rate.

The aim of the present study was to analyze and discuss the available literature data on the incidence of early death and risk factors in pediatric patients suffering from arterial ischemic stroke.

## 2. Methodology

The authors searched PubMed, Scopus, Google Scholar, and Embase using combinations of the following keywords: “arterial ischemic stroke”, “ischemic stroke”, “pediatric stroke”, “neonatal stroke”, “death”, “risk factors”, “children”, “newborns”, and “outcome” (the last search was performed in April 2021).

Articles concerning children (up to 18 years old) with AIS were analyzed. In one of the studies, the upper limit age for pediatric patients was 20 years old [4]. As for the AIS criteria, we followed those described previously by Golomb et al. [12]. In some studies, AIS was discussed together with transient ischemic attack (TIA), thus we also included these papers. We included both perinatal stroke, defined as cerebral circulatory disturbance between the 20th prenatal week to the 28th day of life, as well as neonatal stroke, up to the 28th day of life [3,12]. In turn, data on hemorrhagic stroke and cerebral sinus venous thrombosis (CSVT) were excluded.

Data on the definition of “early death” in pediatric patients are sparse; however, we adopted the term “in-hospital” death (no matter how long the hospitalization related to the stroke was) up to 30 days after the onset of stroke symptoms.

The literature search was limited to studies published from 2000 to 2020.

## 3. Etiology and Clinical Presentation of AIS in Different Pediatric Age Groups

The clinical presentation of pediatric AIS mostly depends on the location of the ischemic focus, the number of infarct foci, and the patient’s age. In older children, the clinical presentation resulting from anterior or posterior brain vascularization can be distinguished. In the former, the most frequent presentation is hemiparesis with central facial nerve palsy accompanied by aphasia if the dominant hemisphere is affected by stroke. According to the number of arteries involved, anterior stroke subtypes are divided into lacunar anterior circulation infarct (LACI), partial anterior circulation infarct (PACI), or total anterior circulation infarct (TACI). If stroke is localized in the posterior brain arterial vasculature, it is called posterior circulation infarct (POCI). In both children and adults, anterior circulation stroke is more common than POCI. The location of AIS is meaningful not only for the clinical presentation in the acute phase of disease, but also in the aspect of prognosis and stroke consequences.

Currently, the most important and most frequently identified risk factors for pediatric AIS include cerebral arteriopathy, mainly focal cerebral arteriopathy of childhood (FCA), followed by congenital and acquired heart disease, conditions conducive to thrombosis, some systemic and metabolic diseases, trauma, and intoxication [13,14,15,16]. FCA is defined as a narrowing of the cerebral vessel, usually unilateral, with a clinically acute and monophasic course unrelated to a specific etiology such as moyamoya disease or syndrome, dissection of the vessel wall, angiopathy following chickenpox, post-radiation vasculopathy, sickle cell anemia, or angiitis [12].

Craniocerebral artery dissection (CCAD) is a problem that mainly affects boys, and accounts for up to 20% of the arteriopathy leading to stroke in children. Usually, as a result of an injury or manipulation in the area of the neck, there is a dissection of the vertebral artery wall, which clinically manifests as headache and hemiparesis. In children, non-traumatic dissections can also be seen, and in these cases, they involve intracranial vessels [17].

A very large group of factors that increase the risk of AIS are those that promote thrombosis, including inborn and unmodified factors (e.g., gene polymorphisms encoding proteins related to the coagulation system) and those that are acquired (e.g., antiphospholipid syndrome). The results of research in this area are inconsistent and sometimes contradictory [18,19], which most often is likely due to the small size of analyzed samples.

As for perinatal stroke, it is defined as a clinical situation when there is a focal disturbance of cerebral circulation secondary to thrombosis or embolization within arterial or venous vessels in the period between the 20th week of fetal life until the 28th day of postnatal life, and the diagnosis is confirmed by the results of neuroimaging or anatomopathological examination [3].

Patients diagnosed with perinatal stroke are divided into two groups depending on the time of symptom onset. In the classic approach, such patients are newborns, considering the neonatal period as the time from birth to the 28th day of life; infants born after 36 weeks of pregnancy are also usually included in the analysis. The neonatal period is characterized by very specific pathophysiology, different from the later period, which results from the permanent and gradually decaying mechanisms of fetal life. The second group comprises patients with so-called presumed perinatal stroke. These are children who did not present acute symptoms in the neonatal period, while the features of a history of stroke begin to become evident, most often in the form of hemiparesis, in the first year of life, and the final diagnosis due to a stroke in the fetal or neonatal period is confirmed based on the results of imaging examinations [20,21].

A prospective 16-year national study in Canada on the epidemiology and risk factors of stroke established the incidence of neonatal stroke at 10.2 new cases per 100,000 live births, which is a significantly higher incidence in the youngest pediatric age group [15]. In turn, in the neonatal period, preterm infants are most frequently affected by stroke, and the rate is 100 new cases per 100,000 births [22].

The clinical presentation of neonatal stroke is also very specific and differs from that seen at a later age. It is dominated by disturbances in consciousness and seizures; this presentation is very nonspecific, thus making the correct diagnosis more difficult.

In a group of 232 Canadian newborns, the vast majority (88%) presented seizures as the predominant symptom of stroke; almost half of them had symptoms described as nonspecific, with no risk factors for stroke. In the case of neonatal stroke, most patients present with permanent motor deficits and a whole range of disorders that significantly affect school achievement, including concentration disorders. Post-stroke epilepsy mainly affects the youngest stroke patients and those diagnosed with FCA and multiple ischemic focus [23,24].

Whereas in the neonatal population, the incidence of stroke is estimated at up to 100 new cases per 100,000 if we include premature newborns, the risk factors for AIS are completely different than in older children, including maternal factors (systemic diseases, hypertension), factors related to the course of labor, and neonatal factors (congenital infections, inherited defects, the need for ECMO, termination of pregnancy by caesarean section or due to forceps or vacuum, perinatal hypoxia, infections, especially neuroinfections, sepsis or hypoglycemia) [25,26,27].

The long-term prognosis after neonatal stroke is unfavorable, because only one-third of children will remain free from neurological deficits, while the majority will present with neurodevelopmental disorders, motor deficits, and epilepsy (up to 50% of patients) [3,28].

## 4. Prevalence of Stroke Mortality

### 4.1. Death in the Early Stage of Ischemic Stroke in the Neonatal Period

The factors for worse prognosis in neonates with stroke are the location of ischemic changes in the area of the basal ganglia, impaired awareness, and nonspecific clinical symptoms in the acute phase. deVeber et al. [15] observed 12 neonatal deaths predischarge (6%), with nearly 42% of them being stroke-specific. Table 1 shows the prevalence of stroke mortality in neonates based on available data.

The results of an international study on hospital deaths in children with stroke, published by Beslow et al. [11], reported that 1.5% of the neonates in the study died. The risk factors for in-hospital death and the time between the first symptoms of the disease and death were established for 9 out of 14 deceased neonates. The median time from onset of stroke symptoms to death in this group was 26 days. In the studied group of newborns, among the symptoms of acute stroke, the size of the stroke focus, i.e., its location in the anterior and posterior cerebral vasculature, also proved to be a factor contributing to an unfavorable prognosis in terms of death [11]. The authors observed that in only two children, death in the hospital was associated only with the AIS itself, while in the remaining seven children, underlying diseases were significant, including congenital heart disease and secondary problems related to AIS such as renal or respiratory failure [11]. In turn, a high frequency of death during the hospital stay was observed by Lee et al. [29], but the study group included only 10 Taiwanese neonates. On the other hand, in a study by Clive et al. [30], no neonates with AIS died within the study period.

### 4.2. Deaths at Early Phase of AIS in Pediatric Population

In pediatric patients aged 28 days to 18 years old, the prevalence of in-hospital mortality differs between studies, as shown in Table 2. The highest percentage of early deaths was observed by Fullerton et al. [4] in a sizeable group of children (almost 1200 cases). However, the upper age limit was set at 20 years old. In addition, the authors demonstrated that boys were found to have a significantly higher risk of death in the first 30 days after stroke (risk ratio (RR) 1.59, 95%, CI 1.18–2.12, *p* = 0.002) [4]. This observation was confirmed by some authors [4,8], whereas others found no such difference [9,11,31,32,33]. A similar high frequency of in-hospital death as in the study by Fullerton et al. [4] was reported by Lopes-Espejo et al. [33] and Chung and Wong [34], accounting for 14% in each study.

An earlier study by Fullerton et al. [9] showed that average annual mortality rate (AAMR) accounted for 0.09/100,000 person-years, while Lehman et al. [35] established AAMR at a level of 0.058/100,000 person-years.

On the other hand, a Polish study based on 89 children and a Turkish study based on 33 children with AIS demonstrated no deaths during the study period [7,36]. An English study on over 200 pediatric patients with AIS demonstrated that 13 died (6%), but provided no information on deaths during the hospital stay [37]. A Taiwanese study demonstrated overall deaths in stroke pediatric patients at 10.5%, but again, without specifying in-hospital deaths [38].

## 5. Risk Factor for Mortality after AIS in Pediatric Patients

### 5.1. Patient’s Ethnicity

A few studies, especially in the past, noted that Black children have a lower stroke-related survival rate than their white peers [9,37]. One of the reasons is sickle cell disease, as it predominantly affects the black population and is a known cause of stroke. Lehman et al. [35] described a drop in stroke mortality rate in a population of Black children in the USA after the Stroke Prevention Trial in Sickle Cell Anemia (STOP trial) was published in 1998. This trial proved that blood transfusion therapy in children with SCD decreased stroke occurrence by more than 90%. As a result, the mortality rate due to stroke declined in the Black population. The authors estimated that the relative risk of stroke mortality for Black vs. white children for the periods 1988 to 1997 and 1998 to 2007 decreased from RR = 1.72, 95% CI 1.27–2.29 to RR = 1.09, 95% CI 0.75–1.54, respectively [35]. This might be one reason why in newer studies, such as a study by IPSS, Black race was found not to be a risk factor for stroke mortality [11].

Hispanic ethnicity is also connected with higher death risk in the pediatric stroke population. This is one possible explanation for the high mortality rate in Chile noted in studies by Lopez-Espejo et al. [31,33]. Hispanic ethnicity also proved to be a risk factor for in-hospital death from stroke in the global population analyzed by IPSS [11]. The explanation for this finding is not clear. However, it could result from some inequalities in health and lower economic status.

Geographical variation was observed in the USA, with higher stroke mortality rates in southeastern states. This phenomenon, already known in adult patient studies as the “stroke belt”, cannot simply be explained by ethnic discrepancies. This location dependence was first described in the pediatric population in a study by Fullerton et al. [42]. Based on data from 1979 to 1998, the authors described a higher risk of death in 11 southeastern vs. other states in the USA (RR = 1.27, 95% CI = 1.11–1.45 *p* = 0.0004) [42]. However, this observation was not confirmed in more recent studies carried out by IPSS and Statler et al. [11,32].

The economic status of the patient’s country is connected with survival chance. Developing countries have a 30- to 60-fold higher absolute number of children’s deaths due to stroke than developed countries. The highest mortality was found in the Caribbean region and central Sub-Saharan Africa, and the lowest in high-income regions of North America, central Latin America, Central Europe and Australasia [10].

### 5.2. Etiopathogenesis and Underlying Diseases

Among conditions that predispose and lead to stroke in children, congenital heart diseases and prothrombotic states were significant in decreasing the long-term survival rate [11,31,33]. A summary of the most important risk factors for stroke mortality by studies with statistical parameters is presented in Table 3. It is also worth noting that the prevalence of some of the underlying conditions depends on ethnicity, with moyamoya disease as one example. In a Taiwanese study by Chiang et al. [38], it had a prevalence of 7.6% in the stroke group, while in a study on the Polish population, there were no cases [7].

The location of the circulation infarct is related not only to stroke symptoms, but also to outcomes in children. Anterior plus posterior stroke in children is a risk factor for death (HR = 2.43, 95% CI 1.42–4.61, *p* = 0.026) [31]. As for posterior/anterior subtypes, the most recent study by Goeggel Simonetti et al. [43] reports the results from the International Stroke Pediatric Study. Based on an analysis of 2768 patients (from neonates to 18 years old), after excluding posterior plus anterior circulation strokes, no statistical difference in mortality was found in the posterior versus anterior circulation ischemic stroke groups. The case fatality rate was equal in both groups: 2.9% (12 out of 419 patients with posterior stroke and 35 out of 1196 patients with anterior stroke) [43].

### 5.3. Other Risk Factors for Death

A study by Lopez-Espejo et al. [31] described a group of 119 children aged 30 days to 18 years with the first incident of cerebral ischemia. Death within the first 30 days of stroke occurred in 14 children (11.7%) and stroke severity was a factor associated with the risk of death in patients over 2 years of age. The severity at baseline was assessed as results obtained in the pediatric NIHSS score and Glasgow scale (<12 points). In the multivariate logistic regression, the authors found that initial stroke severity (assessed by the Pediatric National Institute of Health Stroke Scale (Ped-NIHHSS)) was positively associated with in-hospital mortality (OR 1.11, 95% CI 1.02–1.26, *p* < 0.001) [31].

Brush et al. [40] confirmed that the presence of hypertension within 72 h after stroke occurrence is a risk factor for death, with a risk ratio of 4.5. The number of days the patient had hypertension in the acute phase is also correlated with stroke mortality (*p* = 0.043).

The place of treatment (type of hospital) and the method of treatment may be significant to the outcome of the death of a child with stroke, although the data on this subject are very limited. Statler et al. [32] analyzed the relationship between type of hospital (children’s or non-children’s) and type of care (aggressive care, defined as either systemic pharmacological treatment, catheter-directed therapy, or surgery, or non-aggressive care) and stroke mortality rate in children. No difference was observed for the type of hospital, but higher mortality was observed in the aggressive treatment group. This observation must be interpreted with caution, however, as the authors did not have data on any form of stroke severity assessment and for this particular analysis, patients with both ischemic and hemorrhagic stroke were taken into account [32].

One of the most frequently described causes of death in the early stage of stroke in children and adults is malignant stroke in terms of middle cerebral artery (MCA) vascularization [31].

According to the criteria for the diagnosis of malignant cerebral edema, such a diagnosis can be made if the ischemic obstruction covers more than 50% of the vascularization of the cerebral artery and causes a cerebral edema with a mass effect, and with clinical effect in the form of rapidly progressive deterioration of the patient’s condition and unfavorable prognosis, in terms of both surviving acute stroke and long-term effects [44]. Conservative anti-edema treatment is associated with a death rate up to 80% [45,46]. The results obtained from three randomized European studies conducted in adult patients indicate that almost 80% of patients with malignant MCA edema survive, and about 95% of them present mild or moderate neurological symptoms if surgical treatment by decompressive hemicraniectomy is attempted [45,46,47,48]. In the pediatric population, the incidence of malignant cerebral artery syndrome in the course of stroke is unknown; in a group of 700 children with stroke, the incidence of malignant edema was established at 1.3% [49]. Observations among pediatric AIS patients in 2005 and 2010 in the Greater Cincinnati/northern Kentucky area of 1.3 million inhabitants led to the diagnosis of malignant stroke in two children. One of those children, who underwent hemicraniectomy, survived the acute period of the disease and presented a moderate disability, while a 17-year-old patient with significant disability prior to the onset of stroke did not undergo hemicraniectomy. She died after the acute period of illness [44]. The incidence of malignant stroke, and the incidence of stroke itself, in children, appear to be greatly underestimated but undoubtedly are a risk factor for death in the early phase of the disease; however, the results of individual reports on the effects of hemicraniectomy are very promising.

Age at stroke onset is another possible factor related to survival chances. While newborns and neonates have the highest incidence of ischemic stroke, it is the teenage group (15–19 years old) that proved to have the highest death rate in a study by Krishnamurti et al. [10]. Similar results, i.e., higher risk of death during hospitalization in children (>1 y) than neonates (<1 y), were presented by the International Pediatric Stroke Study (IPSS): OR 2.04 (95%, CI 1.15–3.65, *p* = 0.015) [11]. However, again, in some studies, no difference related to age was found [4].

## 6. Mortality in Children in Relation to Acute-Phase Treatment

While there are clear guidelines and recommendations for treatment in the acute phase of stroke in the adult population, the matter of thrombolytic and endovascular treatment in children remains ambiguous. Currently, there is no consensus on acute treatment strategies or patient inclusion criteria for childhood AIS [3], but as new clinical trials and research results are published, this might change in the near future.

As presented in a study by Marecos et al. [50], one of the main limitations for both the acute therapies is the eligibility of patients. By retrospective analysis of patients with AIS at the Great Ormond Street Hospital in London, it was found that based on criteria of age < 8 years, diagnosis confirmed by CT/MRI within 6 h after symptom onset, lack of contraindications, and occlusion of the major artery seen in imaging methods, out of 107 patients, none could undergo thrombolysis [50]. It is worth noting that the Thrombolysis in Pediatric Stroke (TIPS) prospective study, in which it was planned to give thrombolysis to 48 children above the age of 2 years, was unfortunately closed due to the low number of recruited patients [51]. The protocol assumed criteria of treatment before 4.5 h after symptom onset and CT/MRI confirmation of stroke before treatment, among others. In total, 93 children were screened for TIPS but only 1 was enrolled in the study. The remaining children were excluded for various reasons: stroke mimics, lack of occlusion in arterial imaging, contraindications, too low NIHSS score, or being outside the time window [52].

Studies with data on mortality in patients who underwent thrombolysis [53,54,55,56] are presented in Table 4.

Table 5 presents data on thrombectomy in the pediatric stroke population [57,58,59,60,61,62]. The disparity in mortality rates is significant. After reviewing the literature on the subject of thrombectomy in pediatric stroke, Cappellari et al. [57] concluded that fatal complications may be underreported. One of the interesting differences in the data presented below that may relate to mortality rate is the time between the onset of symptoms and the start of treatment (groin puncture time), but further study is needed.

## 7. Conclusions

Data show that the incidence of in-hospital deaths in children with AIS ranged from 2.6 to 14%, and this spread was mainly due to the different sizes of the studied groups of patients, different risk factors for the occurrence of stroke, and different ages of children enrolled in the study, since a group of newborns was analyzed in the present review. The organization of care for adult patients with AIS and the standards in this area have been functioning for many years in contrast to pediatric patients suffering from AIS. In children, these standards and recommendations for acute stroke care are often used only in preparation. This justifies an in-depth study of groups of children with stroke, optimally homogeneous in terms of age, ethnicity, and geographical location, in order to identify the factors that are the most unfavorable for survival in the acute phase of disease, as the number of early deaths in children could then be decreased.

The interest in the issue of early post-stroke mortality which is analyzed in the present review could generate discussion in the field, which in turn may lead to some solutions in the acute care of pediatric patients with stroke.

## Figures and Tables

**Table 1:** Prevalence of stroke mortality in neonates (age up to 28 days).

Study	Population (Country, Study Period)	Number of Cases	In-Hospital Deaths *N* (%)
Beslow et al. [11]	International Pediatric Stroke Study, 2003–2014	915	14 (1.5)
deVeber et al. [15]	Canada, 1992–2001	201	12 (6) */5 (2.5) **
Lee et al. [29]	Taiwan, 2003–2012	10	1 (10)
Clive et al. [30]	Nova Scotia, Canada, 2007–2013	33	0 (0)

* All deaths predischarge; ** stroke-specific deaths predischarge.

**Table 2:** Prevalence of stroke mortality in pediatric AIS patients.

Study	Population (Age, Country, Study Period)	Number of Cases	In-Hospital Deaths *N* (%)
Fullerton et al. [4]	30 d to <20 y, USA, 1991–2000	1167	192 (16.5)
deVeber et al. [15]	29 d to 18 y, Canada, 1992–2001	576	62 (10.8) */30 (5.2) **
Kopyta et al. [7]	30 d to 18 y, Poland, 2002–2013	89	0 (0)
Beslow et al. [11]	28 d to 19 y, International Pediatric Stroke Study, 2003–2014	2273	70 (3.1)
Lopes-Espejo et al. [31]	30 d to 18 y, Chile, 2003–2015	119	14 (11.8)
Statler et al. [32]	31 d to 19 y, USA, 2000 and 2003	5813	433 (7)
Lopes-Espejo et al. [33]	29 d to 18 y, Chile, 2003–2013	98	14 (14)
Fox et al. [39]	28 d to 19 y, USA, 1993–2003	124	5 (4)
Brush et al. [40]	30 d to 18 y, Australia, 2003–2008	89	7 (8)
Goldenberg et al. [41]	28 d to 19 y, International, 2003–2007	661	22 (3)
Chung and Wong [34]	≤15 y, Hong Kong, 1991–2001	50 ***	7 (14)
Uzunhan et al. [36]	28 d to 18 y, Turkey, 2007–2013	33	0 (0)

* All deaths predischarge; ** stroke-specific deaths predischarge; *** including patients with hemorrhage stroke.

**Table 3:** Risk factors for mortality in children with stroke.

Study	Underlying Conditions Related to Stroke Mortality	OR, HR, or RR (95% CI)	*p*
Fox et al. [39]	Congenital heart disease	HR = 3.62 (1.33–10.93)	0.011
	Prothrombotic state	HR = 3.51 (1.25–9.32)	0.017
	Initial stroke severity	HR = 1.05 (1.01–1.09)	0.017
	Anterior plus posterior stroke	HR = 2.43 (1.42–4.61)	0.026
Lopes-Espejo et al. [33]	Heart disease	OR = 6.57 (1.3–32)	0.020
	Chronic head and neck disease	OR = 41.3 (3.5–490)	0.003
Beslow et al. [11]	Hispanic ethnicity in children >1 y	OR = 3.12 (1.56–6.24)	0.001
	Congenital heart disease (<1 y)	OR =3.88 (1.23–1.22)	0.021
	Congenital heart disease (>1 y)	OR = 3.14 (1.75–5.61)	<0.001
	Anterior plus posterior stroke (<1 y)	OR = 5.36 (1.7–16.85)	0.004
	Anterior plus posterior stroke (>1 y)	OR = 2.71 (1.4–5.25)	0.003
	Stroke presentation without seizures in neonates (<1 y)	OR = 3.95 (1.26–12.37)	0.019
Brush et al. [40]	Hypertension presence in the acute phase after stroke	RR = 4.5 (0.6–34.5)	0.096

RR, risk ratio; OR, odds ratio; HR, hazard ratio; CI, confidence interval.

**Table 4:** Study on thrombolysis in pediatric patients: mortality rates.

Study	Population	Patients Receiving Thrombolysis, *N* (%)	Fatal Cases, *N* (% in Thrombolysis Group)	Overall Mortality in Study (%)
Alshekhlee et al. [53]	9257 patients, Kids’ Inpatient Database	67 (0.7)	2 (10.45)	6.14
Amlie-Lefond et al. [54]	687 patients, IPSS study, Canada and USA	15 (2)	2 (13.3)	3.3
Amlie-Lefond et al. [55]	26 patients, TIPSTER study	26 (100)	0 (0)	-
Janjua et al. [56]	2904 patients, nationwide inpatient sample, USA	46 (1.6)	10 (19.6)	3.4

**Table 5:** Studies on thrombectomy in pediatric population: mortality rate.

Study	Patients, *N* (Age)	Fatal Cases, *N* (%)	Time between Symptom Onset and Treatment (Groin Puncture)
Cappellari et al. [57]	24 (2–17 y)	0 (0)	1.5–72 h
Fragata et al. [58]	7 (2–17 y)	2 (28.6)	Median 7 h 6 min (2 h 58 min to 21 h 38 min)
Bhogal et al. [59]	5 (7–17 y)	0 (0)	Nd
Sun et al. [60]	11 (9 mo to 4 y)	1 (9)	Median 12 h (4–50 h)
Sporns et al. [61]	12 (5–17 y)	0 (0)	Median 2 h (1–4 h)
Sporns et al. [62]	73 (8 mo to 18 y)	1 (1.3)	4 h (IQR 3.0–6.9 h)

nd, no data; IQR, interquartile range.

## Data Availability

Not applicable.
